# Toxic Animal-Based Medicinal Materials Can Be Effective in Treating Endometriosis: A Scoping Review

**DOI:** 10.3390/toxins13020145

**Published:** 2021-02-14

**Authors:** Su-In Hwang, Young-Jin Yoon, Soo-Hyun Sung, Ki-Tae Ha, Jang-Kyung Park

**Affiliations:** 1 Department of Korean Medicine Obstetrics and Gynecology, Pusan National University Korean Medicine Hospital, Yangsan 50612, Korea; hwangsi1216@gmail.com (S.-I.H.); yyj@pusan.ac.kr (Y.-J.Y.); 2 Division of Clinical Medicine, School of Korean Medicine, Pusan National University, Yangsan 50612, Korea; 3 Department of Policy Development, National Development Institute of Korean Medicine, Seoul 04554, Korea; koyote10010@nikom.or.kr; 4 Department of Korean Medical Science, School of Korean Medicine, Pusan National University, Yangsan 50612, Korea; hagis@pusan.ac.kr

**Keywords:** endometriosis, toxic animal-based medicinal materials, Hirudo, Eupolyphaga, Scolopendra, Scorpio

## Abstract

Animal toxins and venoms have recently been developed as cancer treatments possessing tumor cell growth-inhibitory, antiangiogenesis, and proapoptotic effects. Endometriosis is a common benign gynecological disorder in reproductive-age women, and no definite treatment for this disorder is without severe side effects. As endometriosis and malignant tumors share similar characteristics (progressive, invasive, estrogen-dependent growth, and recurrence), animal toxins and venoms are thought to be effective against endometriosis. The objective of this study was to outline studies using toxic animal-based medicinal materials (TMM) as endometriosis treatment and to explore its clinical applicability. Preclinical and clinical studies using TMM were searched for in four databases from inception to October 2020. A total of 20 studies of TMM on endometriosis were included. In eight clinical studies, herbal medicines containing TMM were effective in relieving symptoms of endometriosis, with no side effects. In twelve experimental studies, the main therapeutic mechanisms of TMM against endometriosis were proapoptotic, antiangiogenesis, estrogen level-reducing, and possible anti-inflammatory effects. TMM are thus considered promising sources for the development of an effective treatment method for endometriosis. Further studies are needed to clarify the therapeutic mechanism of TMM against endometriosis and to provide sufficient grounds for clinical application.

## 1. Introduction

Endometriosis is a common estrogen-dependent, chronic inflammatory disease experienced by 10–15% of reproductive-age women [1,2,3]. It is defined as a disease in which endometrium-like tissue exists outside the uterus [2]. Endometriosis negatively affects the quality of life and well-being of women of childbearing age owing to distressing and debilitating symptoms and complications [4]. The most common clinical symptoms are dysmenorrhea, pelvic pain, infertility, and various clinical symptoms, such as dyspareunia, anal pain, and abdominal and adnexal masses [5,6]. Hypotheses on the origin of endometriosis occurrence include endometrial origin due to retrograde menstruation and non-endometrial origin, such as coelomic metaplasia and lymphatic and vascular metastasis [4,7]. The interaction between endocrine, immunologic, pro-inflammatory, and proangiogenic processes appears to be involved in the development of endometriosis [2]. In addition, endometriosis is a benign disease, but it displays similar features to malignancy, such as progressive, invasive, estrogen-dependent growth, and recurrence [8,9].

Conventional treatments for endometriosis include analgesics, combined oral contraceptives, gonadotropin-releasing hormone agonists, and surgery [4,10,11]. However, there are limitations such as unpleasant and sometimes dangerous side effects and high recurrence rates [5]. Thus, patients with endometriosis seek traditional Chinese medicine treatments to cure the primary lesions and relieve symptoms, with fewer side effects [12,13,14]. In traditional Chinese medicine, “blood stasis” is considered the basic etiology of endometriosis, and thus treatment is aimed at removing the stasis by promoting blood circulation [15]. In east Asian countries, animal-based medicinal materials, which eliminate blood stasis and facilitate blood circulation, have traditionally been used to treat diseases. In Korea, there are records that such materials were used to address gynecological diseases caused by blood stasis [16]. It seems that animal-based medicinal materials were used mainly in China, where such substances are widely in use to treat various diseases such as rheumatism [17], kidney disease [18], gynecological diseases [19], and cardiovascular and cerebrovascular diseases [20]. 

Venomous and poisonous animals have long been an area of interest in the treatment of diseases. Animals produce toxins in specialized glands or cells to protect themselves from predators and to predate on other animals [21]. Animal toxins include “poison”, which is produced by specialized cells or tissues and transmitted via ingestion or contact, and “venom”, which is produced in tissues or organs and delivered parenterally via specialized apparatus such as stingers, teeth, and nematocysts. [22]. Animal poisons and venoms are composed of different classes of molecules, and they contain abundant proteins, peptides, and neurotransmitters [21,22]. Although our understanding of the complexity of animal toxins is still limited, recent studies have shown that animal toxins can be developed as cancer treatments because of their ability to inhibit cell growth, inhibit angiogenesis, and induce apoptosis [23,24]. 

Considering the pathophysiology of endometriosis and the treatment mechanism of animal poisons and venoms identified in previous studies, various animal poisons and venoms are thought to be effective against endometriosis. Recently, preclinical and clinical studies investigating the effects of animal poisons and venoms on endometriosis have been reported. However, as far as we know, no review articles on studies using toxic animal-based medicinal materials to treat endometriosis have been published. Therefore, in this study, we outline the use of toxic animal-based medicinal materials for endometriosis and explore the possibility of its clinical use.

## 2. Materials and Methods

### 2.1. Scope of Toxic Animal-Based Medicinal Materials

There are 99 animal-based medicinal materials with seven specifications (Korean Pharmacopoeia, Korean Herbal Pharmacopoeia, Chinese Pharmacopoeia, Japanese Pharmacopoeia, Japanese standards for non-Pharmacopoeial crude drugs, Taiwan Herbal Pharmacopoeia, Democratic People’s Republic of Korea Pharmacopoeia) from five northeast Asian countries [25]. Among them, the following 10 toxic animal-based medicinal materials were selected by identifying toxicity [26]: Vespae Nidus, Tabanus, Mylabris, Agkistrodon, Bufonis Venenum, Hirudo, Scolopendra, Eupolyphaga, Scorpio, and Gecko.

### 2.2. Literature Search Strategy

We searched the following databases from inception to October 2020: MEDLINE, EMBASE, one Chinese database (China National Knowledge Infrastructure (CNKI)), and one Korean database (Oriental Medicine Advanced Searching Integrated System (OASIS)). The search words were “Vespae Nidus OR Nidus Vespae OR Bee venom OR Tabanus OR Mylabris OR Cantharides OR Agkistrodon OR Snake venom OR Bufonis Venenum OR Bufo OR Bufo Siccus OR Toad venom OR Bufonis Crustum OR Toad cake OR Secretio Bufonis OR Hirudo OR Leech OR Scolopendra OR Scolopendrae OR Eupolyphaga OR Steleophaga OR Scorpio OR Gecko OR Tokay” AND “endometriosis”. There were no language limitations in this study. 

### 2.3. Inclusion and Exclusion Criteria

Type of studies: Experimental studies, such as animal or cell studies, and clinical studies using toxic animal-based medicinal materials for treating endometriosis were included in this review. All types of clinical studies, including randomized controlled trials (RCTs), observational studies, cohort studies, case reports, and case series were included in this review. Literature studies, qualitative studies, and surveys were excluded.

Subjects: Clinical studies with female patients with endometriosis were included. Experimental studies on animal endometriosis models or cell culture studies were considered for inclusion.

Types of intervention: Studies using 10 types of toxic animal-based medicinal materials in the treatment of endometriosis patients, endometriosis animal models, and endometriosis cells were included. In the case of toxic animal-based medicinal materials alone, a combination of toxic animal-based medicinal materials and other toxic medicinal materials was also included.

### 2.4. Study Selection and Data Extraction

Two independent authors (SIH and SHS) selected the studies according to the predefined criteria. Any disagreements were discussed by a third author (JKP). Two authors (SIH, GTH) independently extracted the following data from the selected experimental studies: author, year, study design, toxic animal-based medicinal materials used in intervention, target cell (in vitro)/animal model (in vivo), dosage, treatment period, outcome measures, results, and components of herbal medicine. Two authors (SHS, YJY) independently extracted the following data from clinical studies: author, study design, sample size, age, main symptoms, toxic animal-based medicinal materials used in intervention, control intervention, treatment duration, outcome measures, main results, adverse events, components of herbal medicine, dosage, frequency, and dosing period. We resolved any disagreements by discussing with a third author (JKP).

## 3. Results

**Figure 1:**
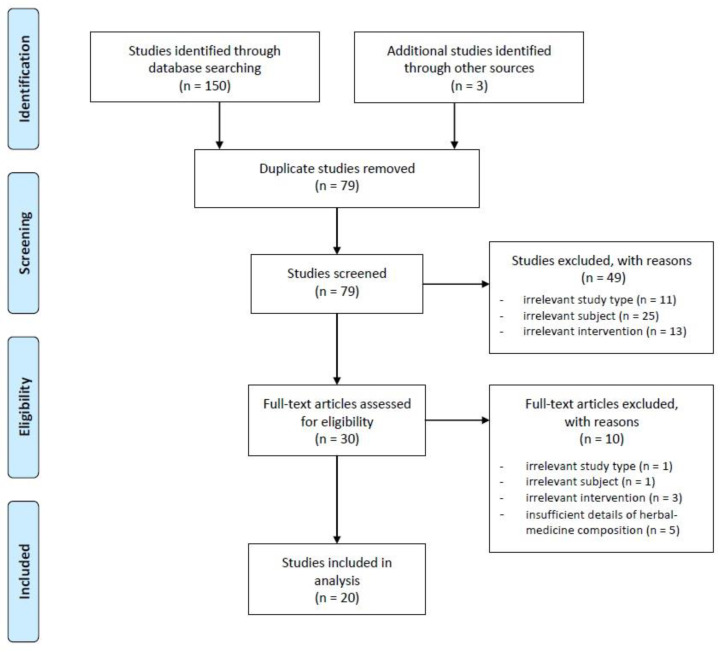
Flow chart of the study selection process. After literature searching, we identified a total of 153 studies, from which 74 duplicate studies were excluded. We screened 79 studies’ titles and abstracts. As a result, 49 studies were excluded, and 30 studies passed the first round of the selection process. Of these 30 studies, 10 irrelevant studies were excluded again, and the final 20 studies were selected for this study. Of the 20 studies finally selected, 8 were experimental studies and 12 were clinical studies.

After literature searching, we identified a total of 153 studies (6 in English database, 146 in Chinese database, and 1 in Korean database), from which 74 duplicate studies were excluded. By screening the titles and abstracts of 79 studies, 11 studies were found to be irrelevant due to the study type (10 reviews and 1 survey), 25 studies irrelevant due to the subject, and 13 studies irrelevant due to the intervention were excluded. After assessing the full text of the remaining 30 studies for eligibility, 20 studies were finally included, excluding one study due to irrelevance in the study type, one study due to irrelevance in the subject, three studies due to irrelevance in the intervention, and five studies due to insufficient details about the composition of the herbal medicine. Of the 20 studies finally selected, 8 were experimental studies and 12 were clinical studies. A flow diagram of the study selection process is shown in Figure 1.

### 3.1. Preclinical Studies 

Among the studies included in this analysis, there were eight preclinical studies [27,28,29,30,31,32,33,34] investigating the effect of animal toxins on endometriosis, two in vitro studies [28,30], one both in vivo and in vitro study [29], and five in vivo studies [27,31,32,33,34].

The medicinal interventions used in the eight studies were largely divided into three categories, namely medicinal compounds extracted from animal toxins in two studies [28,29], individual medicinal extracts in two studies [27,34], and medicinal decoctions in four studies [30,31,32,33]. The composition of the medicinal formulae used in the four studies is summarized in Table 1. 

Among the toxic animal-based medical materials, Hirudo was the most commonly used in medicinal decoctions [30,31,32,33], followed by bufalin twice [28,29]. 

According to the eight studies, the underlying mechanisms of animal toxins against endometriosis were as follows: induction of apoptosis [28,29,30], inhibition of angiogenesis [31,32], reduction of estrogen level [27], suppression of matrix metalloproteinase (MMP)-9 expression [34], and anti-inflammatory effect [33] (Table 2).

### 3.2. Clinical Studies 

In this analysis, we included a total of 12 clinical studies [35,36,37,38,39,40,41,42,43,44,45,46] examining the therapeutic effects of herbal medicine containing animal toxins on endometriosis, of which five were case series [35,36,37,38,39] and seven were randomized controlled trials (RCTs) [40,41,42,43,44,45,46]. According to these studies, dysmenorrhea was the most common symptom in patients diagnosed with endometriosis, followed by infertility, pelvic mass, dyspareunia, pelvic pain, mittelschmerz, and anal pain.

In all the clinical studies, medicinal decoctions were used. Quyu Jiedu Xiaozheng Decoction was used in two studies, but at different doses [38,41]. Other than that, different prescriptions were used in the remaining studies. The toxic animal-based medicinal materials used in these studies include Hirudo, Scolopendra, Eupolyphaga, and Scorpio. Hirudo was used in a total of eight studies [35,36,37,39,40,43,44,46] at daily doses of 0.5 g [37], 1–2 g [43], 4–5 g [40], 6 g [39], 9 g [35], and 10 g [44,46]. Scolopendra was used in five studies [37,38,41,45,46] at varying doses of 0.5 g [37], one to two pieces [36], two pieces [41,46], and 6 g [45] per day. Eupolyphaga was used in four studies [35,38,41,46] at doses of 6 g [38,41], 10 g [46], and 12 g [35] per day. Scorpio was used in one study [42] at a dose of 6 g per day. The administration duration varied from 1 to 9 months, with 3 months [36,37,38,44,45,46] being the most common. The timing of administration also varied; some decoctions were discontinued during the menstrual period [35,39], administered throughout the menstrual cycle [40,42,44,45], or administered after menstruation [37,46].

All included clinical studies reported improvement of symptoms related to endometriosis, and three studies [37,42,45] reported that no side effects were observed (Table 3). The dosing regimens of herbal medicines containing toxic animal-based medicinal materials in the included clinical studies are presented in Table 4.

## 4. Discussion

In this scoping review, we outlined preclinical and clinical studies of toxic animal-based medicinal materials for the treatment of endometriosis. By reviewing the experimental and clinical studies included in this study, we identified the potential of toxic animal-based medicinal materials as a treatment of endometriosis. However, the current evidence is insufficient for establishing a standard for endometriosis treatment with toxic animal-based medicinal materials. High-quality studies are needed to investigate the various action mechanisms of toxic animal-based medical materials against endometriosis.

Even though we did not impose any restrictions in the literature search, we found more studies published in China than in any other country. Traditional Korean medicine and traditional Chinese medicine were alike as both are recognized by the healthcare systems of those counties. However, in the healthcare systems of the two countries, traditional Chinese medicine has a bigger share than that of traditional Korean medicine. Furthermore, traditional Chinese medicine spans a broader range, as it includes not only traditional Chinese medicine but also the integration of Chinese and Western medicine [47]. Therefore, it is believed that there would be more cases of using toxic animal-based medicinal materials in clinical contexts in China, with more reports for that matter.

The toxic animal-based medical materials investigated in the preclinical and clinical studies included in this analysis were somewhat different. Hirudo and Scolopendra were used in both preclinical and clinical studies. In contrast, Eupolyphaga and Scorpio were used only in clinical studies, and bufalin, bee venom, and snake venom were used only in preclinical studies. This is because Bufonis Venenum, bee venom, and snake venom are animal-derived toxins that can cause serious side effects, including death [22,48,49,50,51], and thus have not been traditionally used to treat endometriosis and benign disease. Although these three animal venoms have recently been reported to exert proapoptotic [52,53,54] and anti-inflammatory effects [55,56], there has been no clinical evidence of their benefit in endometriosis treatment. These three venoms are thought to be effective against endometriosis because they have been reported to induce apoptosis, inhibit angiogenesis, reduce estrogen levels, suppress MMP-9 expression, and exert anti-inflammatory effects in preclinical studies. However, considering the serious side effects that may occur, the in vitro and in vivo toxicity and action mechanisms of bufo venom, bee venom, and snake venom must be elucidated before their efficacy against endometriosis can be investigated in clinical trials.

The 12 clinical studies included in this study reported improvement of various endometriosis symptoms, such as dysmenorrhea, pelvic pain, dyspareunia, mittelschmerz, anal pain, as well as increased pregnancy rate after administration of a decoction containing Hirudo, Eupolyphaga, Scolopendra, and Scorpio. However, as there were differences in the symptoms, dosage, and dosing period of the toxic animal-based medicinal materials used, the evidence was considered insufficient for establishing a standard for the application of toxic animal-based medicinal materials in the treatment of endometriosis. In future studies, the optimal dosage of the medicines should be established with consideration for both efficacy and toxicity, and clinical studies must be conducted to standardize the dosing period and dosage.

**Figure 2:**
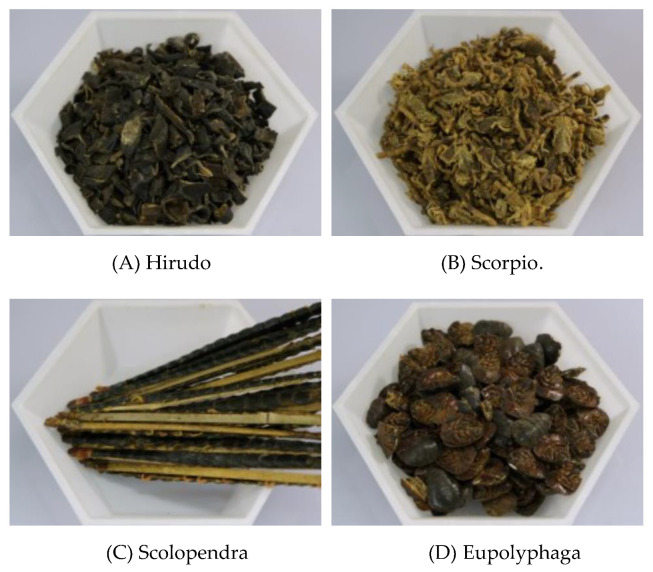
Toxic animal-based medicinal material in the includes clinical studies. (**A**) Hirudo: The dried entire body of *Whitmania pigra* Whitman, *Hirudo nipponica* Whitman, or *W. acranulata* Whitman. (**B**) Scorpio: The dried body of *Buthus martensii* Karsch scorpion. (**C**) Scolopendra: The dried body of *Scolopendra subspinipes mutilans* L. Koch. (**D**) Eupolyphaga: The dried body of female *Eupolyphaga sinensis* Walker or *Steleophaga plancyi* (Boleny).

The most widely used toxic animal-based medicinal material among the included clinical studies was Hirudo Figure 2. Hirudo is the dried entire body of *Whitmania pigra* Whitman, *Hirudo nipponica* Whitman, or *W. acranulata* Whitman, and is a representative toxic animal-based medicinal material that has been used since the beginning of civilization. It has the efficacy of “breaking blood and expelling stasis”, and there is historical evidence of its use in the treatment of endometriosis [22,57,58]. However, it is difficult to confirm the effectiveness of Hirudo against endometriosis, as there were no studies examining only Hirudo. Nevertheless, a medicinal decoction containing Hirudo was shown to exert therapeutic effect against endometriosis in both preclinical and clinical studies. 

In the studies included in this scoping review, medicinal decoctions containing Hirudo was shown to downregulate the expression of hypoxia-inducible factor 1-alpha (HIF-1α) and reduce the level of vascular endothelial growth factor (VEGF), thereby suppressing the progression of endometriosis [32]. They also reduced the levels of basic fibroblast growth factor, platelet-derived growth factor, and VEGF, and were involved in the angiogenesis pathway by downregulating TLR4 and NF-κB, which participate in the signaling process of angiogenesis, thus improving endometriosis [31]. It was also suggested that a medical decoction containing Hirudo exerted anti-inflammatory effect [33]. These findings are supported by other experimental studies. Hirudo has been revealed to have anticoagulant, anti-inflammatory, bacteriostatic, and analgesic effects [22,59]. Hirudo is also known to inhibit tumor angiogenesis by improving the tumor hypoxia microenvironment, reducing the mRNA and protein expression of HIF-1α, and contributing to the downregulation of VEGF mRNA expression [60]. In addition, Hirudo induces apoptosis and inhibits cell proliferation in Hep G2 human liver cancer cells and HL-60 leukemic cells, resulting in an antitumor effect [61,62]. Although the use of Hirudo is controversial, Hirudo exerts anti-inflammatory effect by inhibiting carboxypeptidases (kininase 1), inhibits platelet function, exerts anticoagulant activity, and increases blood flow [63].

Scolopendra was the second-most widely used toxic animal-based medicinal material in the included clinical studies. Scolopendra is the dried body of *Scolopendra subspinipes mutilans* L. Koch, which is poisonous. It has the efficacy of “extinguishing wind and suppressing convulsion, unblocking collaterals and relieving pain, counteracting toxins, and dissipating masses” and is used for “spasm and convulsion due to internal stirring of liver wind, etc.” [57]. Medicinal decoctions containing Scolopendra were shown to be effective in treating endometriosis in both clinical and preclinical studies included in the present review.

Experimental studies included in this study suggested that medical decoctions containing Scolopendra may exert therapeutic effect against endometriosis, which is a chronic inflammatory condition, through anti-inflammatory action [33]. This is supported by other studies, in which Scolopendra was reported to have anticoagulation, antiseptic, and anti-inflammatory effects [64,65,66]. Scolopendra is also known to induce the downregulation of matrix metallopeptidase-2 (MMP-2) and -9 in tumor cells, suppress the migration and invasion of tumor cells, and inhibit cell proliferation [67]. In addition, Scolopendra inhibits the proliferation of EGFR-overexpressing cells by inducing apoptosis and modulating the EGFR pathway [68,69]. It also exerts anti-inflammatory activity partially through inhibition of the NF-κB signaling pathway [70].

Eupolyphaga, the third-most commonly used toxic animal-based medicinal material in this review, is the dried body of female *Eupolyphaga sinensis* Walker or *Steleophaga plancyi* (Boleny), which is slightly poisonous. It has the efficacy of “breaking blood and expelling stasis” and has been used clinically for “aggregation-accumulation due to static blood obstruction” [57]. Clinical studies in this review revealed that medicinal decoctions containing Eupolyphaga were effective in alleviating endometriosis symptoms. However, there have been no experimental studies that elucidate the anti-endometriosis mechanism of Eupolyphaga. Nevertheless, in other studies, Eupolyphaga was shown to inhibit cell adhesion to fibronectin and collagen IV as well as cell migration and invasion in A549 human lung cancer cell [71]. It also inhibits cell proliferation and reduces MMP-2 and -9 expression in hepatocellular carcinoma [72], and reduces cell invasive ability by downregulating MMP-2 and -9 protein expression in breast cancer [73]. Furthermore, it induces the detachment and apoptosis of A549 human lung cancer cells [71], inhibits tumor cell growth in hepatocarcinoma, promotes TNF-α and IFN-γ expression, increases the Bax/Bcl-2 ratio, and activates caspases-3 to induce apoptosis [74]. Moreover, Eupolyphaga is known to activate the immune function by modulating oxidative systems, enhancing the phagocytic function of macrophages, and elevating serum IL-2 level [75]. 

Lastly, the dried body of *Buthus martensii* Karsch scorpion is known to be poisonous and exert liver-pacifying and wind-extinguishing effects. Scorpio has pharmacological properties, such as pain-reducing, anti-inflammatory, and anticoagulant properties [76,77], but no experimental studies have determined the therapeutic mechanism of Scorpio against endometriosis. Scorpio exerts an antitumor effect by inhibiting cell proliferation, inducing apoptosis, and decreasing migration and invasive functions in Hepa 1–6 cells and HepG2 cells [78,79]. They also inhibit MMP activity in breast and colorectal cancers, thereby reducing the motility and invasion of tumor cells, and induce apoptosis by decreasing the expression of antiapoptotic proteins and increasing that of proapoptotic proteins [80]. In addition, chlorotoxin derived from scorpion toxin inhibits the expression of ERα in breast cancer cells by inhibiting the ERα signaling pathway through direct binding to ERα, thus changing the protein secondary structure of its LBD domain [81].

**Figure 3:**
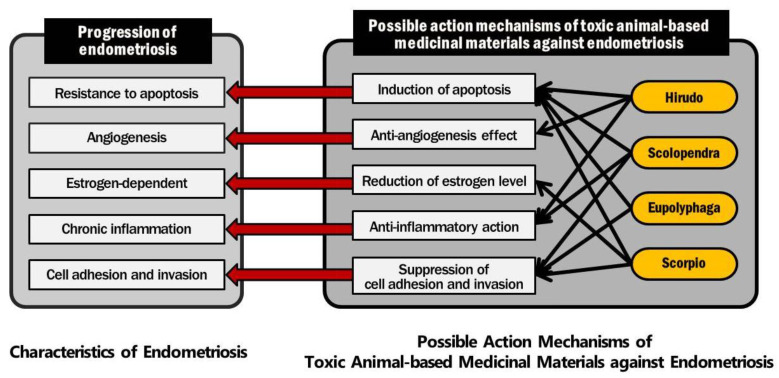
Possible action mechanisms of toxic animal-based medicinal materials against endometriosis.

As outlined above, toxic animal-based medicinal materials are composed of several molecules that exhibit a wide range of pharmacological activities. Considering that endometriosis has several possible mechanisms of pathogenesis, multi-target medicines have the potential to treat endometriosis through multiple pathways. Further research is needed to elucidate the specific therapeutic mechanisms of individual toxic animal-based medicinal materials against endometriosis. Although the mechanisms underlying the progression of endometriosis are still unclear, endocrine, immunologic, pro-inflammatory, and pro-angiogenic processes are known to be involved in the development of endometriosis. Considering the pharmacological effects of toxic animal-based medicinal materials on endometriosis and other diseases, the possible action mechanisms of toxic animal-based medicinal materials against endometriosis can be summarized as follows: induction of apoptosis, antiangiogenesis effect, reduction of estrogen level, anti-inflammatory action, and suppression of cell adhesion and invasion (Figure 3).

Toxic animal-based medicinal materials may cause unwanted side effects due to their toxic effects. Minimizing adverse events is an essential part of drug development. Even with the medicines that have been used based on historical experience, the types and frequency of adverse events must be identified. However, among the studies included in this review, 75% of the studies had no reports on side-effects, and three studies [37,42,45] reported that no side effects were observed after administration of herbal medicines containing Hirudo and Scolopendra [37], Scolopendra [45], and Scorpio [42]. However, it was not possible to find any information on what kind of events they were monitoring. 

The number of participants who participated in the studies that reported no side effects was small. Since the known occurrence of adverse events was 5% to 10% [82,83], the findings of this study are not sufficient to support a conclusion that the administration of toxic animal-based medicinal materials upon the patients with endometriosis would result in no side effects. Even if the toxic animal-based medicinal materials have historical evidence, it is necessary to identify the existence, occurrence rate, and severity of adverse events through real data collection. A clinical study must be designed to report any adverse events, no matter how insignificant they may look.

In addition, in order to identify the possible adverse events that can be caused by toxic animal-based medicinal materials, we searched other studies that reported adverse events after administering toxic animal-based medicinal materials. Among the studies included in this review, three studies [37,42,45] reported that no side effects were observed after administration of herbal medicines containing Hirudo and Scolopendra [37], Scolopendra [45], and Scorpio [42], and that no adverse effects were reported following the administration of herbal medicines containing Eupolyphaga. However, these findings are inconsistent with those of other studies. Hirudo is prohibited for pregnant women, patients with profuse menstruation or hemorrhagic tendency, patients with no blood stasis, and weak patients because Hirudo is highly potent in breaking blood and expelling stasis, unblocking the meridian, and eliminating mass [26,57]. Zeng et al. reported gastrointestinal bleeding (1.9%) and cerebral hernia (0.9%) after the administration of herbal medicines containing Hirudo and *Tabanus bivittatus* Matsumura in patients with acute intracerebral hemorrhage [84]. Historically, owing to its toxicity, Scolopendra has not been used at a high dosage, as its excessive use can damage healthy qi and cause early abortion [26]; therefore, it is prohibited for use in pregnant women and patients with blood deficiency generating wind [57]. In one patient with chronic hepatitis B infection, hepatotoxicity was reported after the administration of herbal medicine containing Scolopendra [85]. However, this herbal medicine contained not only Scolopendra but also the hepatotoxic Meliae Fructus and Rhei Radix et Rhizoma [85]; these toxic effects cannot be attributed solely to Scolopendra, but caution is needed when subjecting patients with chronic hepatitis B to Scolopendra treatment. In traditional Chinese medicine, Eupolyphaga is contraindicated for pregnant women because it may induce early abortion owing to its blood-breaking and stasis- expelling efficacies, and it should be used with caution in patients with no blood stasis or patients with both blood deficiency and blood stasis [26]. Several studies have reported the side effects of Eupolyphaga. However, in a study where 21 HIV/AIDS patients were treated with a drug containing 20 g of Eupolyphaga twice a day for 4 months, no side effects or toxic reactions were reported [86]. Scorpion should be prohibited in patients with blood deficiency generating wind, and should be used at a dose range of 3–5 g owing to its toxicity [26,57]. There was one case of hepatotoxicity after administration of herbal medicine containing Scorpio in a patient with chronic hepatitis B infection [85]. Taken together, the patient’s conditions should be considered when administering toxic animal-based medicinal materials, and attention should be paid to the menstrual cycle during treatment with these materials, as they can cause excessive bleeding in patients with menorrhagia. In addition, when administering these medicinal materials to women of childbearing age, the possibility of pregnancy should be assessed. Moreover, overuse of these medicinal materials should be avoided because it has been reported to be associated with hepatitis.

This is the first study to review both preclinical and clinical studies investigating the efficacy of toxic animal-based medicinal materials in the treatment of endometriosis. Our analysis results suggested the possible therapeutic mechanisms of toxic animal-based medical materials against endometriosis and provided data as a reference for future translational studies. In addition, we included studies from major databases, such as PubMed and Embase, and searched without language and date restrictions. However, there were some limitations in this review. First, as we used only the biopharmaceutical names as search terms, there is a possibility that potentially relevant articles not containing our search key words and phrases in the titles or abstracts were not included in our analysis. Second, the number of included studies was small. Third, it was difficult to determine whether the reported therapeutic effects were exerted by the toxic animal-based medicinal materials, as these materials were used concomitantly with other medicinal materials. Fourth, there were some factors that required attention in interpreting the study findings. Five of the clinical studies we reviewed were case series, and therefore the level of evidence was somewhat low. Seven RCTs did not provide their protocols and were without any description of their randomization or blinding methods. Furthermore, some studies used mifepristone or different types of herbal medicines rather than the conventional treatment of endometriosis as control, so caution is needed as one interprets the results of these studies. Some studies did not report side effects or what kind of important harms or unintended effects they observed. Therefore, even if some studies did not report side effects, it does not necessarily support the conclusion that toxic animal-based medicinal materials are safe to use. Lastly, endometriosis is diagnosed by ascertaining symptoms, undertaking examination, and performing laparoscopic surgery [87]. Of the studies that were included in this review, only three [32,34,35] used laparoscopes to diagnose endometriosis. Other studies all relied on presumed diagnosis based on symptoms, examination, ultrasonography, and lab test results, without any classification of endometriosis, which were the limitations of these studies.

Through this scoping review, we showed that toxic animal-based medicinal materials might be effective as a clinical treatment of endometriosis. However, because the number of clinical studies included was small and the intervention and treatment goals were different, the findings of this review are insufficient for establishing a standard for the application of toxic animal-based medicinal materials as a treatment of endometriosis. In addition, although toxic animal-based medicinal materials have been shown to be effective against endometriosis in experimental studies, research on their specific therapeutic mechanisms is still lacking. Therefore, evidence supporting the use of toxic animal-based medicinal materials as a treatment of endometriosis is still limited, and further research is necessary.

The preclinical studies and clinical studies included in this review showed that the most promising toxic animal-based medicinal material for endometriosis is likely to be Hirudo. As shown in Figure 3, Hirudo, Scolopendra, and Scorpio meet three categories of action mechanisms related to the progression of endometriosis. Eupolyphaga meets two categories. In addition, in the clinical studies shown in Table 3, Hirudo was included in eight studies. Scolopendra, Eupolyphaga, and Scoprpio were included in five, four, and one study, respectively. Thus, we assume that Hirudo might be the most prominent candidate for treating endometriosis, among these toxic animal-based medicinal materials. The preclinical studies demonstrated that Hirudo has activities inducing apoptosis and inhibiting angiogenesis and inflammation. Although these molecular mechanisms are closely related to the progression of endometriosis, the anti-endometriotic efficacy of Hirudo was not fully demonstrated by a clinical study. In addition, there were no studies that used Hirudo only, so further study is needed to examine the treatment effect of Hirudo upon endometriosis. Scorpio, Scolopendra, and Eupholyphaga have been in clinical use, but their preclinical study is not sufficient. Bee venom, Bufonis Venenum, and snake venom have been used in preclinical contexts, without any report of toxicity. As bee venom, Bufonis Venenum, and snake venom may cause a serious adverse event, it is necessary to identify the adverse events and appropriate doses via preclinical studies.

In the future, the following steps would have to be taken in order to develop a new drug for endometriosis based on toxic animal-based medicinal materials. First, classical literature and medical records should be searched to obtain additional evidence supporting the use of toxic animal-based medicinal materials in clinical setting. Next, extensive preclinical studies are needed to elucidate the mechanisms of endometriosis treatment that are frequently used in clinical practice. Research is needed not only on the therapeutic mechanism of a single toxic animal-based medicinal material against endometriosis but also on the interaction between medicinal materials and decoctions that are traditionally used in combinations, including toxic animal-based medicinal materials. As they have toxic properties, quality control of single toxic animal-based medicinal materials is necessary, and it is important to assess their safety and toxicity in future studies. Additional clinical trials should be conducted to establish the standardized as well as optimal dosage and dosing period of these medicinal materials.

## 5. Conclusions

In this scoping review, preclinical and clinical studies investigating the efficacy of toxic animal-based medicinal materials against endometriosis did not provide sufficient evidence to allow standardization of these materials as a treatment of endometriosis. However, the included studies have identified toxic animal-based medical materials as potential treatments for endometriosis. In clinical studies, the administration of herbal medicines containing toxic animal-based medicinal materials alleviated symptoms such as dysmenorrhea, infertility, endometrioma, and menstrual irregularities in patients with endometriosis, with no side effects. In experimental studies, the main action mechanisms of toxic animal-based medicinal materials against endometriosis were identified as induction of apoptosis, antiangiogenesis effect, reduction of estrogen levels, and possibly, anti-inflammatory action. Taken together, toxic animal-based medicinal materials are considered promising to be developed as an effective treatment of endometriosis; however, additional studies are needed.

## Figures and Tables

**Table 1:** Composition of the herbal medicines used in the included preclinical studies.

First Author (year)	Herbal Medicine	Component
Liu (2014) [30]	Pingchongjiangnifang	Cinnamomi Ramulus 15 g, Draconis Sanguis 3 g, Trogopterorum Faeces 10 g, Typhae Pollen 6 g, Aquilariae Lignum 6 g, Hirudo 3 g, Liriopis seu Ophiopogonis Tuber 10 g, Glycyrrhizae Radix et Rhizoma 6 g
Xu (2018) [31]	Eleng Capsule	Curcumae Rhizoma 10 g, Sparganii Rhizoma 10 g, Salviae Miltiorrhizae Radix 15 g, Curcumae Radix 15 g, Paeoniae Radix Rubra 15 g, Pelodiscis Carapax 15 g, Hirudo 3 g
Liu (2020) [32]	Huangzhi Neiyi Capsule	Hirudo 3 g, Rhei Radix et Rhizoma 9 g, Cyperi Rhizoma 6 g
Wang (2005) [33]	Wudan Wan Modified Decoction	Salviae Miltiorrhizae Radix 10 g, Paeoniae Radix Rubra 10 g, Curcumae Rhizoma 10 g, Cinnamomi Cortex 6 g, Corydalis Tuber 10 g, Hirudo 10 g, Scolopendra two pieces, Pelodiscis Carapax 15 g, Sappan Lignum 10 g

**Table 2:** Characteristics of the included preclinical studies.

Study ID	Author (Year)	Study Design	Herbal Medicine (Toxic Animal-Based Medicinal Materials)	Target Cells (In Vitro)/Animal Model (In Vivo)	Dosage	Treatment Period	Outcome Measures	Results
1	Lee (2006) [27]	In vivo	Bee venom (n.s.)	Female SD rats (autotransplantation method)	0.1 mg/mL (0.1 mL, CV4)	6 weeks (3 times/week)	1) Histological analysis (H&E) 2) Serum progesterone 3) Serum estradiol 4) Serum cytokines (TNF-α, IL-2, -4, -6, -10)	1) Inhibited proliferation of endometrial tissue 2) NSD 3) Decreased ^a^ 4) TNF-α, IL-4, NSDIL-2: Decreased ^a^ IL-6, IL-10: Increased ^a^
2	Nasu (2005) [28]	In vitro	Bufalin (Bufonis Venenum)	Human endometrial cells	0, 0.001, 0.01, 0.1, 1, 10 ng/mL	-	1) Cell proliferation and cell viability 2) Apoptotic cells(%) 3) Cell cycle assay(flow cytometry) 4) Apoptosis-related proteins and cell cycle-related proteins	1) Decreased ^e^ 2) Increased ^d^ 3) G0/G1 cell cycle arrest observed ^c^ 4) Downregulated(Cyclin A, Bcl-2, Bcl-XL proteins), Upregulated(Bax, p21, cleaved caspase-9 protein), Unchanged(Cyclin B, cyclin D3, Fas, Fas ligand, cleaved caspase-3, cleaved caspase-8 protein)
3	Cho (2018) [29]	In vivo	Bufalin (Bufonis Venenum)	Female C57BL/6 mice (Autotransplantation method)	1 mg/kg	21 days	1) Volume of endometriotic lesions 2) Protein levels of the SRC-1 isoform 3) Protein levels of ERβ	1) Decreased ^b^ 2) Increased ^b^ 3) Decreased ^a^
		In vitro		Human endometrial cells	0, 10, 20, 50, 100, 200, 400, 800 nM	-	1) Intrinsic transcriptional activity of the SRC-1 isoform 2) Intrinsic transcriptional activity of ERβ3) Levels of PSMD2 4) Levels of Ki-67 5) Levels of active caspase 3 6) Levels of active IL-1β 7) Levels of caspase 1 8) Levels of ER-stress markers(PERK, PDI, and BiP)	1) Increased ^b^ 2) NSD 3) Increased (epithelium ^d^, stroma ^d^) 4) Decreased (stroma ^c^) 5) Increased (epithelium ^a^) 6) Increased (stroma ^d^) 7) Increased (stroma ^c^) 8) PERK: Increased (epithelium ^d^, stroma ^d^), PDI: Increased (epithelium ^c^, stroma ^d^), BiP: Increased (epithelium ^c^, stroma ^a^)
4	Liu (2014) [30]	In vitro	Pingchongjiangnifang(Hirudo)	Human endometrial cells	5%, 10%, 15%, 20% of the drug in serum (1 mL)	-	1) Cell proliferation 2) Number of adherent cells 3) Number of invading cells 4) Apoptosis rate	1) Decreased ^a^ 2) Decreased ^a^ 3) Decreased ^a^ 4) Decreased ^b^
5	Xu (2018) [31]	In vivo	Eleng Capsule(Hirudo)	Female SD rats (autotransplantation method)	4 g/kg (high dose), 2 g/kg (medium dose), 1 g/kg (low dose)	28 days	1) Volume of endometriotic foci 2) Histological analysis (H&E) 3) Serum levels of VEGF 4) Serum levels of bFGF 5) Serum levels of PDGF 6) Expression of VEGF 7) Expression of bFGF 8) Expression of PDGF 9) Expression of NF-κB 10) Expression of TLR4	1) Decreased (high ^a^/medium ^a^/low ^a^) 2) Improved 3) Decreased (high ^b^/medium ^b^/low ^b^) 4) Decreased (high ^b^/medium ^b^/low ^b^) 5) Decreased (high ^b^/medium ^a^/low ^a^) 6) Decreased (high ^b^/medium ^b^/low ^b^) 7) Decreased (high ^b^/medium ^b^/low ^b^) 8) Decreased (high ^b^/medium ^b^/low ^b^) 9) Decreased (high ^b^/medium ^b^/low ^b^) 10) Decreased (high ^b^/medium ^b^/low ^b^)
6	Liu (2020) [32]	In vivo	Huangzhi Neiyi Capsule (Hirudo)	Female SD rats (autotransplantation method)	9 g/kg (high dose), 4.5 g/kg (low dose)	28 days	1) PCNA 2) CD31 3) VEGF in peritoneal fluid 4) mRNA expression of VEGF 5) mRNA expression of HIF-1α	1) Decreased ^a^ 2) Decreased ^a^ 2) Decreased (high ^a^/low ^b^) 3) Decreased (high ^b^/low ^b^), 4) Decreased (high ^b^/low ^a^)
7	Wang (2005) [33]	In vivo	Wudan Wan Modified Decoction (Hirudo, Scolopendra)	Female SD rats (autotransplantation method)	1 mL/100 g (26.56 g/kg) (high dose), 1/2 diluted (medium dose), 1/4 diluted (low dose)	28 days	1) TER for disappearance of ectopic cysts 2) Effects on abdominal inflammation 3) TER for ectopic endometrial atrophy	1) Improved (medium ^a^/high ^a^), NSD (low) 2) Less severe adhesion (medium/high), More severe adhesion (low) 3) Improved (medium ^b^/high ^a^), NSD (low)
8	Zhang (2015) [34]	In vivo	*Gloydius brevicaudus* venom (Snake venom)	Female SD rats (autotransplantation method)	0.75 mg/kg (low dose), 1.5 mg/kg (high dose)	3 weeks	1) Volume of ectopic foci 2) Expression of MMP-9	1) Decreased ^a^ 2) Decreased ^a^
		In vitro		Rat ectopic endometrial cells	Disintegrin 1.5 µg/mL	-	1) Expression of MMP-9	1) Decreased ^b^

^a^*p* < 0.05; ^b^ *p* < 0.01; ^c^ *p* < 0.001; ^d^ *p* < 0.0001. SD rats: Sprague–Dawley rats; n.s.: not stated; n.r.: not reported; TER: total effectiveness rate; mo: month; NSD: no significant difference; SRC-1: steroid receptor coactivator-1; ERβ: estrogen receptor beta; PSMD2: proteasome 26S subunit, non-ATPase 2; IL: interleukin; PERK: protein kinase RNA-like endoplasmic reticulum kinase; PDI: protein disulfide isomerase; BiP: binding immunoglobulin protein; H&E: hematoxylin and eosin staining; MMP-9: matrix metallopeptidase-9; VEGF: vascular endothelial growth factor; bFGF: basic fibroblast growth factor; PDGF: platelet-derived growth factor; NF-κB: nuclear factor kappa-light-chain-enhancer of activated B cells; TLR4: toll-like receptor 4; PCNA: proliferating cell nuclear antigen; CD31: cluster of differentiation 31; HIF-1α: hypoxia-inducible factor 1-alpha.

**Table 3:** Characteristics of the included clinical studies.

Study ID	Author (Year)	Study Design	Sample Size (TG/CG)	Mean Age ± SD (Min, Max)	Main Symptoms	Diagnostic Criteria	Intervention	Control Intervention	Treatment Period	Outcome Measures	Main Results	AE
1	Wu (1993) [35]	Case series	60	n.r. (20,48)	Dysmenorrhea, infertility, menstrual disorder	Symptom, gynecological exam, ultrasonography	Herbal medicine	-	n.r.	1) TER	91.67%	n.r.
2	Zhang (2007) [36]	Case series	30	32 (23,43)	Dysmenorrhea, mittelschmerz, endometrioma	Symptom, gynecological exam, ultrasonography	Herbal medicine	-	3 mo	VAS	Positive^a^	n.r.
3	Xu (2007) [37]	Case series	52	n.r. (23,46)	Dysmenorrhea, pelvic pain, anal pain, dyspareunia, menstrual disorder, infertility	Symptom, gynecological exam, ultrasonography	Herbal medicine	-	3 mo	1) TER	88.46%	none
4	Zhang (2007) [38]	Case series	78	34.5 (21,46)	Dysmenorrhea, pelvic mass, infertility	Gynecological exam, ultrasonography, laparoscopy	Herbal medicine	-	3 mo	1) TER for dysmenorrhea 2) TER for pelvic mass 3) Pregnancy rate (within 2 years)	1) 94.11% 2) 87.5% 3) 19.05%	n.r.
5	Feng (2014) [39]	Case series	35	n.r. (23,42)	Infertility	Symptom, gynecological exam, ultrasonography	Herbal medicine	-	1–6 mo	1) TER for pregnancy 2) Spontaneous abortion rate in early pregnancy	1) 82.86% 2) 17.14%	n.r.
6	Han (2009) [40]	RCT	156 (78/78)	28.5 (18,48)	n.r.	Symptom, laparoscopy	Herbal medicine(A)	Herbal medicine(B)	9 mo	1) TER 2) Pregnancy rate 3) Recurrence rate	1) Positive ^b^ 2) 11.53%(TG) vs 0%(CG) 3) Positive ^b^	n.r.
7	Zhang (2009) [41]	RCT	128 (45/43/40)	n.r. (n.r.)	Dysmenorrhea, pelvic mass	Symptom, gynecological exam, ultrasonography, laparoscopy, antiendometrial antibody, CA125	Herbal medicine(A)	CG1: Herbal medicine(B) CG2: Herbal medicine(C)	3–6 mo	1) TER 2) Dysmenorrhea score 3) Pelvic mass score	1) Positive (vs CG1 ^a^ /CG2 ^a^) 2) Positive (vs CG1 ^b^ /CG2 ^b^) 3) Positive (vs CG1 ^b^ /CG2 ^b^)	n.r.
8	Lin (2011) [42]	RCT	70 (40/30)	n.r. (n.r.)	Dysmenorrhea	Symptom, ultrasonography, CA125	Herbal medicine	Mifepristone	6 mo	1) TER 2) TER for dysmenorrhea 3) CA125	1) NSD 2) Positive ^a^ 3) Positive ^a^	none
9	Meng (2012) [43]	RCT	312 (156/156)	28.5 (18,48)	n.r.	Symptom, gynecological exam, ultrasonography	Herbal medicine(A)+Control intervention	Herbal medicine(B)	9 mo	1) TER 2) Pregnancy rate 3) Recurrence rate	1) Positive ^b^ 2) 11.54%(TG) vs 0%(CG) 3) Positive ^b^	n.r.
10	Guo (2013) [44]	RCT	94 (48/46)	TG: 32.0±9.6 (22,42) CG: 31.0±7.8 (23,40)	Infertility	Symptom, gynecological exam, ultrasonograph, CA125	Herbal medicine +Control intervention	Desogestrel and Ethinyl Estradiol	3 mo	Pregnancy rate	Positive ^b^	n.r.
11	Liu (2017) [45]	RCT	72 (36/36)	n.r. (24,45)	Dysmenorrhea	Symptom, gynecological exam, ultrasonography	Herbal medicine	Sanjie Zhentong Capsule	3 mo	1) TER 2) VAS	1) Positive ^a^ 2) Positive ^a^	none
12	Yi (2018) [46]	RCT	60 (30/30)	TG: 29.41±4.7 (n.r.) CG: 30.0±4.1(n.r.)	Dysmenorrhea, dyspareunia	Symptom, ultrasonography, antiendometrial antibody, CA125	Herbal medicine +Control intervention	Gestrinone	3 mo	1) TER 2) VAS	1) Positive ^a^ 2) Positive ^a^	n.r.

^a^*p* < 0.05; ^b^ *p* < 0.01; ^c^ *p* < 0.001. TG: treatment group; CG: control group; SD: standard deviation; min: minimum; max: maximum; AE: Adverse event; n.r.: not reported; TER: total effectiveness rate; mo: month; VAS: visual analog scale; RCT: randomized controlled trial; NSD: no significant difference; CA125: cancer antigen 125. Positive means that there was an improvement in the outcome. A lower VAS, recurrence rate, dysmenorrhea score, pelvic mass score, and CA124 meant an improvement with the status of endometriosis, and a higher TER indicated a better condition.

**Table 4:** Dosing regimens of herbal medicines containing toxic animal-based medicinal materials in the included clinical studies.

Study ID	Author (Year)	Herbal Medicine		Component	Toxic Animal-Based Medicinal Materials	Dosage, Frequency	Dosing Period
1	Wu (1993) [35]	n.r.		Sparganii Rhizoma 9 g, Curcumae Rhizoma 8 g, Liquidambaris Fructus 9 g, Hirudo 9 g, Paeoniae Radix Rubra 9 g, Manitis Squama 12 g, Eupolyphaga 12 g, Moutan Radicis Cortex 12 g, Salviae Miltiorrhizae Radix 12 g, Cyperi Rhizoma 12 g, Prunellae Spica 12 g	Eupolyphaga, Hirudo	n.r.	Medication discontinued during menstruation
2	Zhang (2007) [36]	n.r.		Salviae Miltiorrhizae Radix, Persicae Semen, Corydalis Tuber, Curcumae Rhizoma, Hirudo, Linderae Radix, Olibanum, Myrrha, Cinnamomi Cortex	Hirudo	One dose per day, bid	n.r.
3	Xu (2007) [37]	Leech Tongluo Capsule (Shuizhi tongluo jiaonang)		Hirudo 0.5 g, Manitis Squama 0.5 g, Astragali Radix 0.5 g, Draconis Sanguis 0.5 g, Codonopsis Pilosulae Radix 0.5 g, Sparganii Rhizoma 0.5 g, Curcumae Rhizoma 0.5 g, Scolopendra 0.5 g, Cnidii Rhizoma 0.5 g, Glycyrrhizae Radix et Rhizoma 0.5 g	Hirudo, Scolopendra	Six granules/time, tid	From MCD 5
4	Zhang (2007) [38]	Quyu Jiedu Xiaozheng Decoction (Quyu Jiedu Xiaozheng tang)		Sargentodoxa cuneata 15–30 g, Hedyotidis Herba 15–30 g, Astragali Radix 30 g, Coicis Semen 30 g, Scapharcae seu Tegillarcae Concha 15 g, Litchi Semen 15 g, Cremastrae Tuber 9–12 g, Draconis Sanguis 6–9 g, Olibanum 6–9 g, Myrrha 6–9 g, Eupolyphaga 6 g, Scolopendra 1–2 pieces, Agastachis Herba 9 g, Glycyrrhizae Radix et Rhizoma 6 g	Eupolyphaga, Scolopendra	One dose per day, n.r.	n.r.
5	Feng (2014) [39]	Xiaoyi Zhuyun tang		Angelicae Gigantis Radix 12 g, Paeoniae Radix Rubra 12 g, Paeoniae Radix Alba 12 g, Cyperi Rhizoma 12 g, Morindae Radix 12 g, Patriniae Radix 12 g, Sparganii Rhizoma 20 g, Curcumae Rhizoma 20 g, Astragali Radix 20 g, Galli Gigeriae Endothelium Corneum 20 g, Gleditsiae Spina 20 g, Lycopi Herba 20 g, Dipsaci Radix 20 g, Drynariae Rhizoma 20 g, Eucommiae Cortex 20 g, Coicis Semen 20 g, Bupleuri Radix 9 g, Hirudo 6 g, Cervi Cornu 10 g	Hirudo	One dose per day, bid	From MCD 6 to MCD 15 (10 days)
6	Han (2009) [40]	A	n.r.	Hirudo 4–5 g	Hirudo	n.r., bid	From 3–4 days before menstruation to the end of menstruation
		B	n.r.	Salviae Miltiorrhizae Radix 10–15 g, Angelicae Gigantis Radix 8–10 g, Salvia chinensis Benth 8–10 g, Myrrha 10 g, Sparganii Rhizoma 8 g, Leonuri Herba 8 g			
7	Zhang (2009) [41]	Quyu Jiedu Xiaozheng Decoction (Quyu Jiedu Xiaozheng tang)		Sargentodoxa cuneata 30 g, Hedyotidis Herba 30 g, Coicis Semen 30 g, Astragali Radix 30 g, Scapharcae seu Tegillarcae Concha 15 g, Litchi Semen 15 g, Cremastrae Tuber 10 g, Agastachis Herba 9 g, Scolopendra two pieces, Draconis Sanguis 6 g, Eupolyphaga 6 g, Olibanum 6 g, Myrrha 6 g, Glycyrrhizae Radix et Rhizoma 6 g	Eupolyphaga, Scolopendra	One dose per day, bid	n.r.
8	Lin (2011) [42]	Whole scorpion bergamot powder (Quanxie Foshou san)		Scorpio 6 g, Angelicae Gigantis Radix 15 g, Cnidii Rhizoma 10 g, Leonuri Herba 15 g, Cyperi Rhizoma 15 g	Scorpio	One dose per day, n.r.	From 3–4 days before menstruation
9	Meng (2012) [43]	A	n.r.	Hirudo 1–2 g	Hirudo	n.r., tid	From 3–4 days before menstruation to the end of menstruation
		B	n.r.	Angelicae Gigantis Radix 10–15 g, Cnidii Rhizoma 6–10 g, Leonuri Herba 15 g, Paeoniae Radix Rubra 10 g, Paeoniae Radix Alba 10 g, Curcumae Longae Rhizoma 10 g, Sparganii Rhizoma 10 g, Curcumae Rhizoma 10 g, Sargentodoxa cuneata 30 g, Sepiae Endoconcha 12 g			
10	Guo (2013) [44]	Bushen Quyu Decoction (Bushen Quyu tang)		Lycii Fructus 15 g, Rehmanniae Radix Preparata 15 g, Ligustri Lucidi Fructus 15 g, Cuscutae Semen 15 g, Angelicae Gigantis Radix 10 g, Paeoniae Radix Rubra 15 g, Paeoniae Radix Alba 15 g, Achyranthis Radix 12 g, Bupleuri Radix 10 g, Sappan Lignum 10 g, Sparganii Rhizoma 10 g, Curcumae Rhizoma 10 g, Hirudo 10 g	Hirudo	One dose per day, bid	From MCD 1
11	Liu (2017) [45]	Tongjingxiao Granules (Tongjingxiao keli)		Salviae Miltiorrhizae Radix 20 g, Paeoniae Radix Rubra 20 g, Angelicae Gigantis Radix 10 g, Scolopendra 6 g, Corydalis Tuber 20 g, Meliae Fructus 10 g, Glycyrrhizae Radix et Rhizoma 6 g	Scolopendra	One dose per day, bid	From 7 days before menstruation to the end of menstruation
12	Yi (2018) [46]	Xiaoyi Analgesic Soup (Xiaoyi Zhitong tang)		Cinnamomi Ramulus 20 g, Poria Sclerotium 30 g, Salviae Miltiorrhizae Radix 20 g, Persicae Semen 20 g, Curcumae Rhizoma 10 g, Scolopendra two pieces, Eupolyphaga 10 g, Hirudo 10 g, Pelodiscis Carapax 20 g	Eupolyphaga, Hirudo, Scolopendra	n.r., bid	From the end of menstruation

n.r., not reported; bid, twice a day; tid, three times a day; MCD, menstrual cycle day.

## Data Availability

The data will be made available upon reasonable request.
